# Striatal dopamine transporter SPECT quantification: head-to-head comparison between two three-dimensional automatic tools

**DOI:** 10.1186/s13550-020-00727-w

**Published:** 2020-11-07

**Authors:** Silvia Morbelli, Dario Arnaldi, Eugenia Cella, Stefano Raffa, Maria Isabella Donegani, Selene Capitanio, Federico Massa, Alberto Miceli, Laura Filippi, Andrea Chincarini, Flavio Nobili

**Affiliations:** 1 Department of Health Science (DISSAL), University of Genoa, Genoa, Italy; 2 IRCCS Ospedale Policlinico San Martino, Genoa, Italy; 3 Department of Neuroscience (DINOGMI), University of Genoa, Genoa, Italy; 4 Genoa Section, National Institute of Nuclear Physics (INFN), Genoa, Italy

**Keywords:** Dopamine transporter, SPECT, Parkinson, Datquant®, BasGanV2, Semi-quantification

## Abstract

**Purpose:**

Our aim was to compare a widely distributed commercial tool with an older free software (i) one another, (ii) with a clinical motor score, (iii) versus reading by experts.

**Procedures:**

We analyzed consecutive scans from one-hundred and fifty-one outpatients submitted to brain DAT SPECT for a suspected parkinsonism. Images were post-processed using a commercial (Datquant®) and a free (BasGanV2) software. Reading by expert was the gold standard. A subset of patients with pathological or borderline scan was evaluated with the clinical Unified Parkinson’s Disease Rating Scale, motor part (MDS-UPDRS-III).

**Results:**

SBR, putamen-to-caudate (P/C) ratio, and both P and C asymmetries were highly correlated between the two software with Pearson’s ‘*r*’ correlation coefficients ranging from .706 to .887. Correlation coefficients with the MDS-UPDRS III score were higher with caudate than with putamen SBR values with both software, and in general higher with BasGanV2 than with Datquant®. Datquant® correspondence with expert reading was 84.1% (94.0% by additionally considering the P/C ratio as a further index). BasGanV2 correspondence with expert reading was 80.8% (86.1% by additionally considering the P/C ratio).

**Conclusions:**

Both Datquant® and BasGanV2 work reasonably well and similarly one another in semi-quantification of DAT SPECT. Both tools have their own strength and pitfalls that must be known in detail by users in order to obtain the best help in visual reading and reporting of DAT SPECT.

## Introduction

Dopamine transporter (DAT) brain SPECT is widely used to diagnose parkinsonian disorders. Image reading is currently the recommended way of reporting [1] but semi-quantification with automatic, three-dimensional tools can reliably assist visual reading, especially in doubtful or borderline cases [2]. Several methods have been proposed, some available either free [3] or commercial [4], with results varying mainly depending upon region of interest (ROI) identification method [5]. However, seldom head-to-head comparisons between such tools have been performed [5, 6], or attempts have been made to comparatively correlated data with a clinical measure as an ‘external’ reference.

Our aim was to compare a widely distributed commercial and tool with an older free software (1) one another, (2) with a clinical motor score, (3) versus reading by experts.

## Materials and methods

One-hundred and fifty-one consecutive patients (87 males, mean age: 72.5 ± 9.2, range 46–82) submitted to brain DAT SPECT for suspected degenerative parkinsonism in a clinical setting were enrolled in a single center using a 2-head, parallel-hole, high-resolution collimator camera (Discovery®, G.E. Healthcare, Hatfield, Hertfordshire, UK). Patients received intravenously 150–185 Mbq I-123 Ioflupane (Datscan®, G.E. Healthcare, as above) and were scanned for 40 min between 3 and 5 h after injection, according to the European Association of Nuclear Medicine guidelines [1]. Images were reconstructed on the Xeleris® workstation using an Ordered Subset-Expectation Maximization algorithm (10 subset, 10 iterations) with a 0.6 Butterworth filter, and corrected for attenuation with the Chang method (coefficient 0.11 cm^−1^).

Patients were informed that their images could have been used for retrospective research purposes and gave their written consent for usage and publication in an anonymized form.

Reconstructed images were visually analyzed by two experts independently, blind to clinical information, who agreed to classify 136 (90%) scans. The remaining 10% was resolved by a 3^rd^ expert. At last, there were 79 positive, 56 negative, and 16 borderline scans. Images were then automatically processed by Datquant® (G.E. Healthcare, as above) and by BasGanV2 [3] (freely downloadable from https://www.aimn.it/site/page/gds/gds-5). The two software automatically position three-dimensional ROI and allow to compute specific-to-non-displaceable binding ratio (SBR) by normalizing counts on an occipital ROI, and to compare values with a group of control subjects embedded in the software itself. Datquant® automatically reorients images and recognizes the reconstruction procedure adapting control subjects to the one under examination. Moreover, it computes SBR for anterior and posterior putamen separately. On the other hand, BasGanV2 requires manual image re-orientation, does not distinguish anterior and posterior putamen, and performs partial volume effect (PVE) correction. A detailed description of the method followed to achieve PVE correction can be found in the original paper describing and validating the BasGan algorithm [7].

The four comparison steps were (1) correlation analysis between SBR of the four basal ganglia, the putamen/caudate ratio of each side, the caudate and the putamen asymmetry, as obtained by the two software; (2) Bland–Altman analysis to assess systematic bias, limits of agreement, and proportional bias between the two methods; (3) correlation analysis between these eight values and the Movement Disorder Society-Unified Parkinson’s Disease Rating Scale, motor section (MDS-UPDRS-III) in the subset of patients with a positive or borderline scan on expert reading and an available MDS-UPDRS-III score (49 patients). Datquant® yielded a positive result in 37 cases (75.5%), was borderline in 3 (6.1%), and negative in 9 (18.4%). On the other hand, BasGanV2 was positive in 39 patients (79.6%), borderline in 8 (16.3%), and negative in 2 (4.1%). The full concordance between Datquant® and BasGanV2 was in 40 patients (81.6%). At the end of diagnostic procedure, 33 patients were diagnosed with Parkinson’s disease, 5 with dementia with Lewy bodies, 3 with corticobasal syndrome, 2 with idiopathic REM sleep behavior disorder, 1 with progressive supranuclear palsy, 1 with frontotemporal dementia, 2 with tremor of unknown origin, and 2 with unspecified dementia); (4) comparison with reading by experts as the gold standard. The software output could be negative, positive, or borderline (if falling between 1.64 and 2.17 standard deviation below the average value, adjusted for age). For discrepant cases, the putamen-to-caudate (P/C) ratio was regarded as a further index of normalcy/pathology with reference to specific normal cutoff. The lower P/C limits were 0.79 in the right and 0.77 in the left hemisphere, respectively, for Datquant® [8]; they ranged between 0.763 and 0.815 for BasGanV2, according to age [3]. All correlation analyses were corrected (Bonferroni) for multiple comparisons.

## Results

**Fig. 1:**
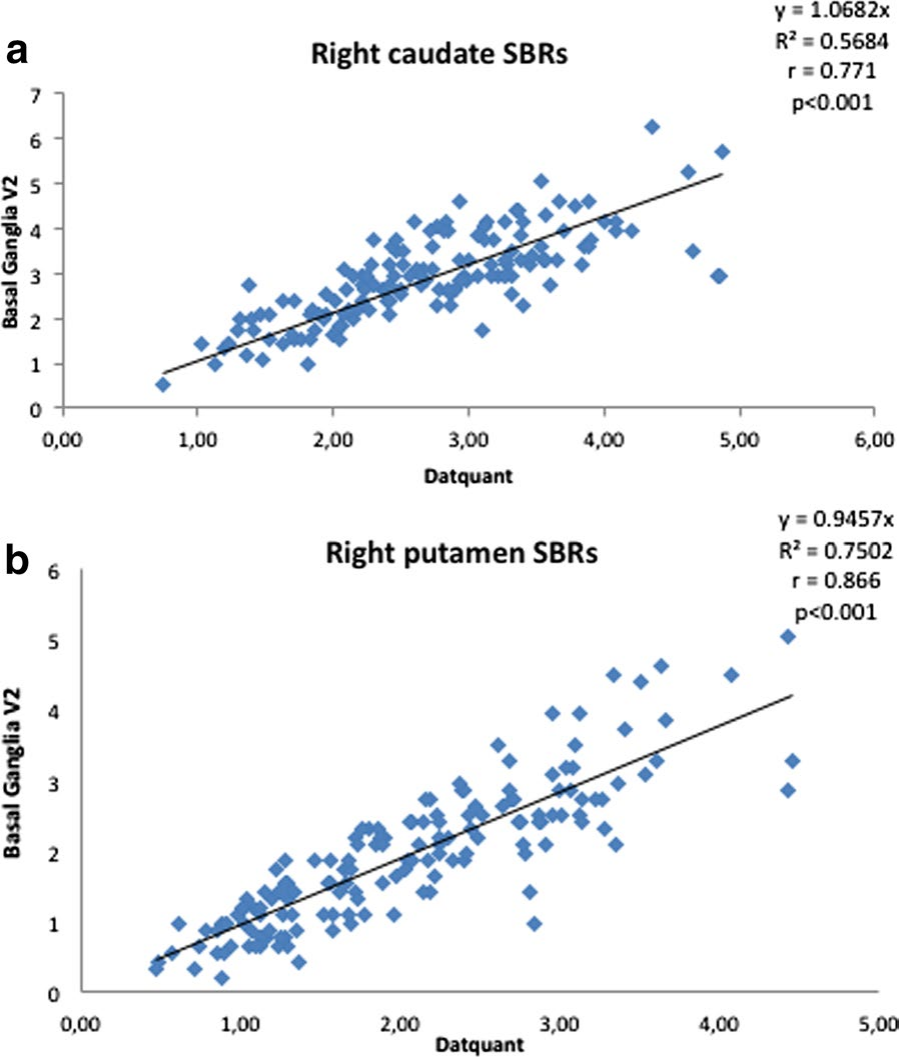
Plot of linear correlation between the SBR of the caudate nucleus (**a**) and of the putamen (**b**) as achieved with Datquant® (*x*-axis) and BasGanV2 (*y*-axis) in 151 subjects. Intercept equation, *R*^2^, *r*, and *p* values are embedded in the figures

SBR, P/C ratio, and asymmetries were significantly correlated between the two software (Table 1) with correlation coefficients ranging from *r* = 0.706 (left P/C ratio) to *r* = 0.887 (caudate SBR asymmetry) (Fig. 1).

**Table 1:** (a) Correlation between Datquant® and BasGanV2 SBR, ratio, and asymmetries and (b) correlation between MDS-UPDRS-III score and SBR, ratio, asymmetries achieved with Datquant® and BasGanV2

	Right caudate	Left caudate	Right putamen	Left putamen	Right P/C ratio	Left P/C ratio	Caudate asymmetry	Putamen asymmetry
(a)								
Datquant®/ BasGan V2	*r* = *.771* *p* < .0001*	*r* = *.752* *p* < .0001*	*r* = *.866* *p* < .0001*	*r* = *.847* *p* < .0001*	*r* = *.793* *p* < .0001*	*r* = *.706* *p* < .0001*	*r* = *.887* *p* < .0001*	*r* = *.783* *p* < .0001*
(b)								
Datquant® /UPDRS-III	* − .414* *p* = .0015*	* − .434* *p* = .0009*	* − .251* *p* = *.0405*	* − .282* *p* = *.0248*	*.027* *p* = *.4275*	*.095* *p* = *.2573*	*.423* *p* = .0012*	*.201* *p* = *.0413*
BasGan V2 /UPDRS-III	* − .523* *p* = .0001*	* − .532* *p* = .0001*	* − .366* *p* = .0048*	* − .447* *p* = .0006*	* − .361* *p* = .0053*	.082 *p* = .2884	*.277* *p* = *.0268*	*.321* *p* = *.0122*

In the columns, the values of SBR, P/C ratio, and asymmetry are reported. Asymmetries are computed without taking into account the side of the more affected hemisphere. See the text for abbreviations. Italics characters show statistically significant correlation (one-tail Pearson’s *r*), while * denotes those correlations surviving Bonferroni’s correction for multiple comparisons (*p* = .00625 as first level of statistical significance)

**Fig. 2:**
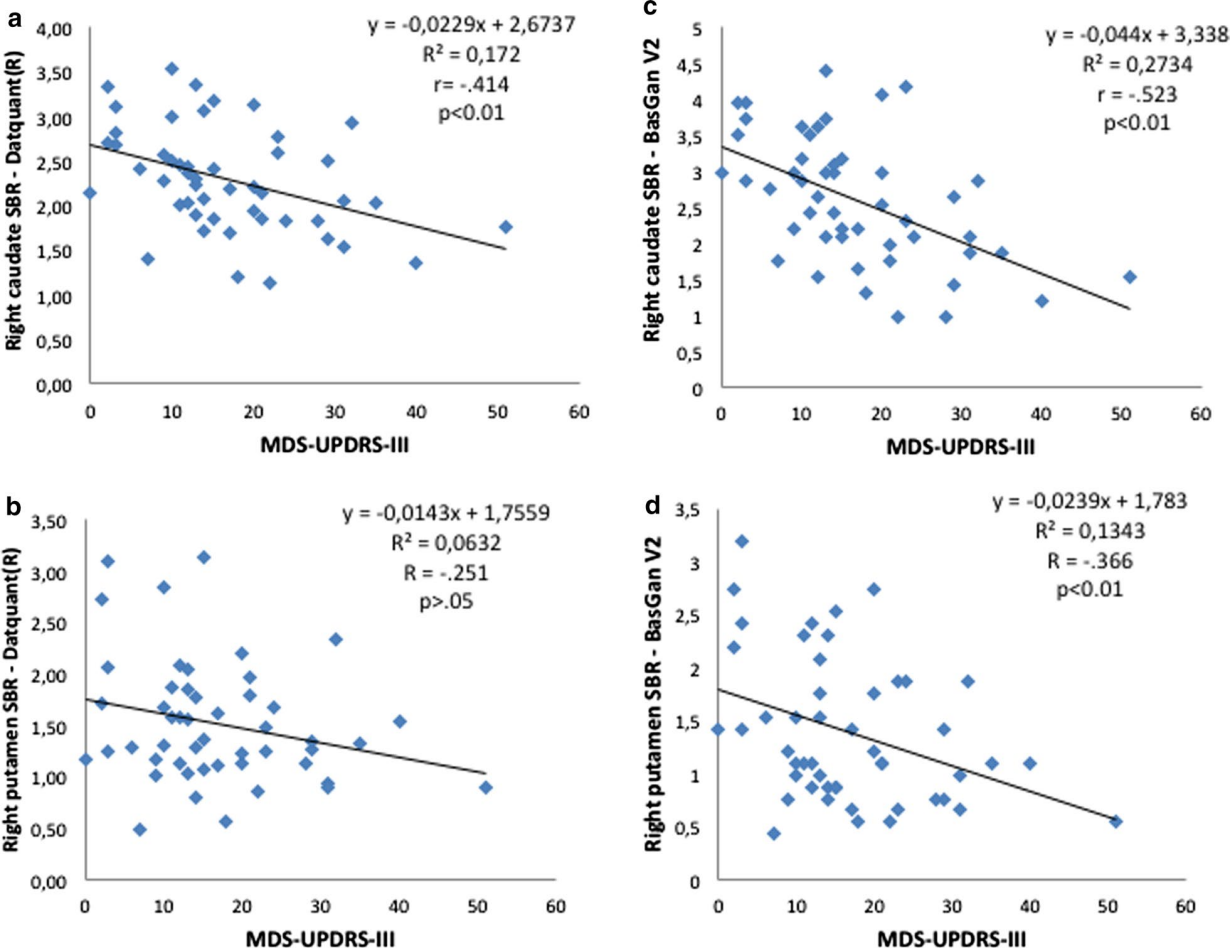
Plot of linear correlation between MDS-UPDRS-III score (*x*-axis) and the SBR of the caudate and putamen nuclei as achieved with Datquant® (**a**, **b**) and BasGanV2 (**c**, **d**) in 49 patients with parkinsonian syndromes. Intercept equation, *R*^2^, *r*, and *p* values are embedded in the figures

Correlation coefficients with the MDS-UPDRS-III score were higher with caudate than with putamen SBR values with both software, and in general higher with BasGanV2 (Table 1; Fig. 2).

Datquant® semi-quantification correspondence with expert reading was in 127 (84.1%) instances. Discrepancies included twelve patients with a major mismatch, i.e., an altered scan according to experts was normal on Datquant®, eleven patients with a borderline scan according to experts but normal on Datquant®, and one patient with a borderline scan for experts but an altered scan on Datquant®. By considering the P/C ratio as a further index of abnormality, ten out of the twelve patients who were normal on Datquant® scored lower than the established cutoff. This raises the agreement with experts to 137 (90.7%) instances.

BasGanV2 semi-quantification correspondence with expert reading was in 122 (80.8%) instances. Discrepancies included only two major disagreements, in which BasGanV2 gave normal SBR despite abnormal expert reading, or *vice versa*. The majority of disagreement concerned normal expert reading with BasGanV2 borderline results (20 instances). The remaining seven discrepancies included a combination of positive by experts/BasGanV2 borderline (3 instances), borderline by experts/BasGanV2 positive (3 instances), and borderline by experts/BasGanV2 negative (1 instance). If we consider the P/C ratio as a further index of abnormality, the only false negative scan showed an abnormally low P/C ratio, 5 out of 20 normal scans for experts but borderline with BasGanV2 had indeed normal P/C ratios, and two more abnormal cases for experts with borderline values on BasGanV2 had indeed pathological P/C ratio values, raising correspondence with experts to 130 (86.1%).

**Fig. 3:**
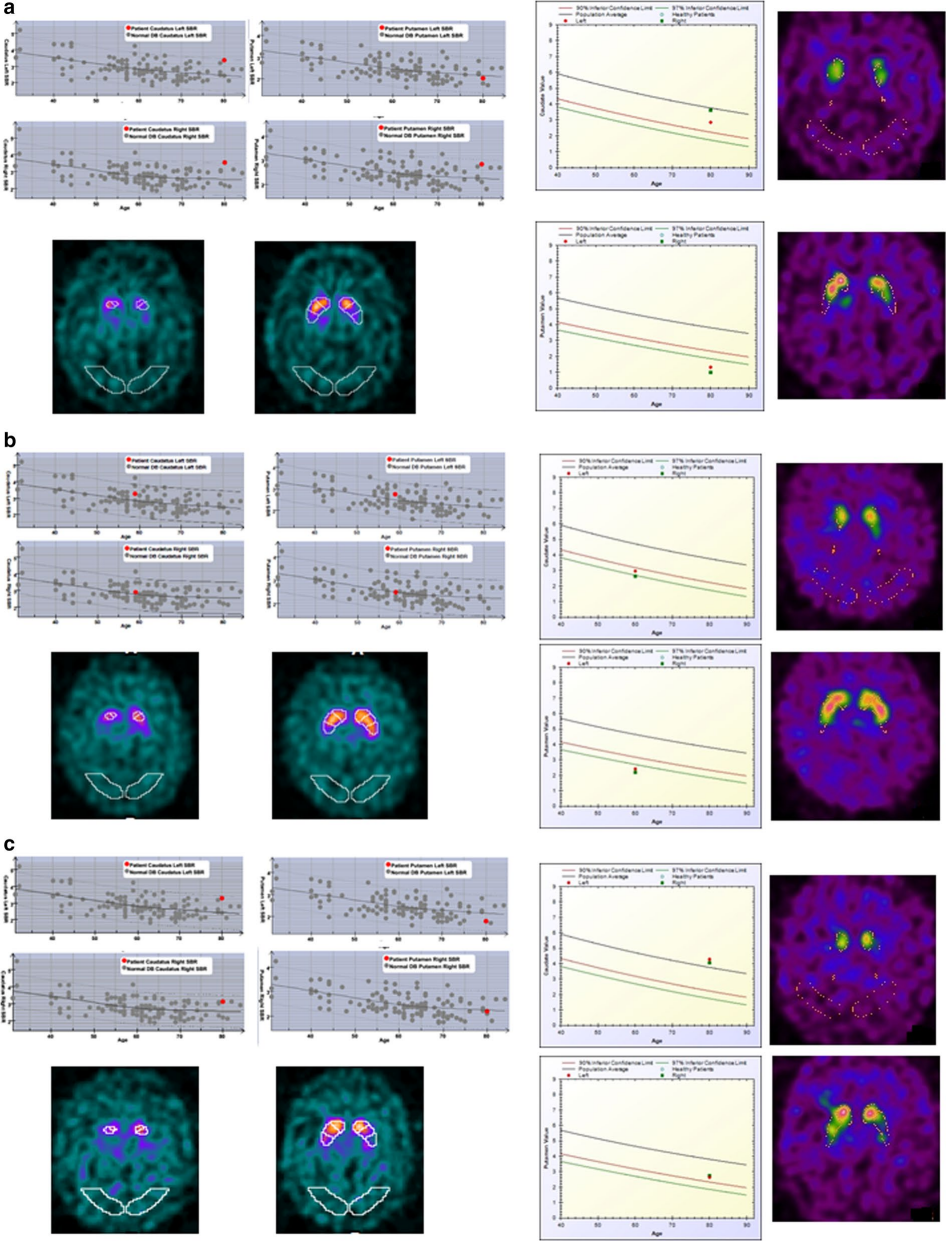
Examples of extreme discrepant results between the two software (**a**, **b**) and between the expert reading and either of the two software (**c**). **a** A 80-year-old man with de novo Parkinson’s disease presenting with bradykinesia, right hand resting tremor and upper arm rigidity, mainly on the right side. The diagnosis is confirmed at 1-y follow-up visit with moderate response to L-DOPA therapy. On DAT SPECT, the experts report reduced tracer uptake in both putamina, mainly on the left side, consistent with clinical presentation. BasGanV2 (upper right) identifies normal caudate uptake but significantly reduced putamen uptake bilaterally. This finding is missed by Datquant® that even shows SBR values at the upper limit of normalcy (upper left). The two images in the lower part of **a** are examples of ROI drawing on the basal ganglia and background, the latter partially falling outside the brain in the right image. Of note, Datquant® correctly highlights a reduced ratio between putamen and caudate uptake (z-score − 3.47 on the left side) thus confirming that the bias is introduced by the background ROI. On left, for Datquant®, the position of patient SBR (*y*-axis) is shown in red in the graph, while gray points report values in normal subjects and the gray lines represent the mean with upper and lower limits of normal distribution, respectively, according to age (*x*-axis). This is shown for each of the four nuclei On right, for BasGanV2, the patient SBRs of either the two caudate nuclei or the two putamen are reported together, the right side in green and the left side in red; the black line represents the mean of normal controls with respect to age (*x*-axis), while the red and the green line report the 90% and 97% confidence level of the normal distribution, respectively. The two images on right are examples of ROI drawing on the basal ganglia and background. **b** A 60-year-old man with essential tremor. The diagnosis is confirmed at 1-y follow-up visit with moderate response to propranolol therapy. On DAT SPECT, the experts report a normal scan. This is confirmed by Datquant® analysis (mid-left), whereas BasGanV2 (mid-right) identifies borderline SBR values for caudate and significantly reduced SBR for putamen bilaterally. Other details as in **a**. **c** A 80-year-old man with de novo Parkinson’s disease presenting with bradykinesia, both resting and intention tremor at upper arms, with prevalence on the right side, constipation and REM sleep behavior disorder. The diagnosis is confirmed at 1-y follow-up visit with good response to L-DOPA therapy. On DAT SPECT, the experts report reduced tracer uptake in both putamina, mainly on the left side, consistent with clinical diagnosis. On the contrary, both Datquant® (bottom left) and BasGanV2 (bottom right) identify normal caudate and putamen SBRs. Other details as in **a**

Of note, in four instances (2.6%), the two software were concordantly against the expert reading, including two negative cases for the experts but borderline for the two software, one borderline case but positive for the two software, and one positive case but negative for the two software. Representative examples of discordant results between Datquant® and BasGanV2 using expert reading as gold standard and reporting the final diagnosis are reported in Fig. 3.

**Fig. 4:**
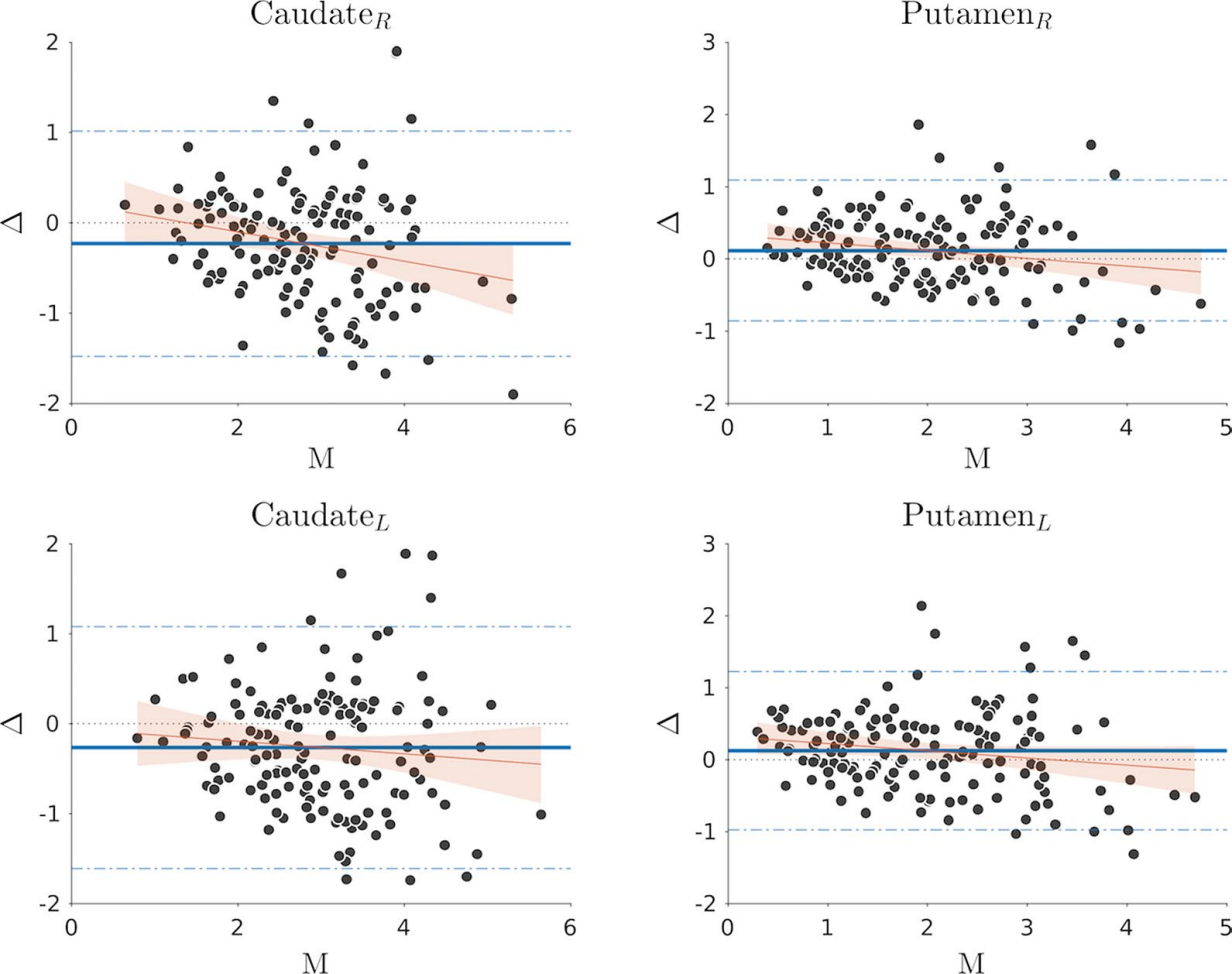
Bland–Altman plot showing the mean bias between Datquant® and BasGanV2 SBR values for each of the four basal ganglia nuclei, with the 95% confidence limits (CL). Proportional bias regression line and its CLs. Numerical results for all variables are reported in Table 2

The Bland–Altman plot (Fig. 4) shows the mean bias between Datquant® and BasGanV2 SBR values (computed as Datquant® minus BasGanV2 SBRs) for each of the four basal ganglia nuclei, with the 95% confidence limits (CL), as well as proportional bias regression line and its CLs. Numerical results for all variables are reported in Table 2. The analysis shows significant proportional bias for the right caudate and the two putamina, not significant for the left caudate, and the difference always decreases in proportion to the average values. This means that BasGanV2 SBR values are higher when the nuclei mean SBR are higher (more preserved) or, equivalently, Datquant® SBR values are lower, and vice versa when the nuclei mean SBR are lower.

**Table 2:** Results of Bland–Altman analysis

	Caudate, R	Caudate, L	Putamen, R	Putamen, L
Bias	− 0.231	− 0.264	0.117	0.126
Bias SD	0.636	0.687	0.498	0.561
Bias SE	0.052	0.056	0.040	0.046
Bias CLs	− 1.478; 1.016	− 1.609; 1.082	− 0.858; 1.092	− 0.975; 1.226
Proportional	− 0.162	− 0.070	− 0.108	− 0.100
Bias (a)				
Proportional	− 0.278; − 0.046	− 0.192; 0.051	− 0.195; − 0.022	− 0.193; − 0.007
Bias (a), CLs				
Proportional	0.221	0.054	0.333	0.323
Bias (b)				
Proportional	− 0.118; 0.560	− 0.432; 0.324	0.143; 0.522	0.119; 0.528
Bias (b), CLs				

(a) and (b) = values of (a) and (b) of fitting equation; *Y* = *ax* + *b* of the proportional bias

SD, standard deviation; SE, standard error; CL, confidence limits, L, left; R, Right

## Discussion

We have shown that two software for DAT SPECT semi-quantification are highly correlated one another both in absolute SBR and derived P/C ratio and asymmetry values. Highest correlation values were reached for the caudate asymmetry and the putamen SBR. However, although the bias between the two methods was near zero in all the four basal ganglia, the Bland–Altman analysis showed large bias confidence limits and, overall, significant proportional bias for all the nuclei but the left caudate. The proportional bias showed that BasGanV2 SBRs are higher than Datquant® SBR in more preserved nuclei while are lower in more damaged nuclei, rendering a more extended value range between maximum and minimum SBRs. Reasons for such bias are likely the different principles underlying the automatic ROI positioning with the two methods and possibly the PVE correction embedded in BasGanV2. Thus, even though the two methods produce highly correlated values, the biases between them are significant so that they cannot be used interchangeably.

The correlation coefficients with the MDS-UPDRS-III (motor) score were generally significant with both tools but they were higher with BasGanV2 than with Datquant® and, with both tools, more at the caudate than at the putamen level. Correlation between this clinical score and basal ganglia was reported to be similar for caudate and putamen [9], higher for the caudate [10], or the putamen [11], likely depending on the semi-quantification tool and on the patient population. Although the nigro-putaminal impairment should be ideally better correlated to a clinical motor score, the identification of caudate nucleus by automatic software could be more accurate than the putamen because of the very low putamen uptake in severely ill patients. Moreover, the BasGanV2 software includes PVE correction, while Datquant® does not; thus, as a speculation, we may suppose PVE correction has allowed the better correlation achieved with the former, although we cannot demonstrate such an effect because the BasGanV2 software does not allow SBR computation without concomitant PVE correction.

Correspondence with expert reading was good (> 80% for both software) especially if the P/C ratio was taken into account in those cases showing discrepancies between experts and software, raising the correspondence with experts of 5.3% and 6.6% for BasGanV2 and Datquant®, respectively (thus to 86.1% and 90.7%). Thus, correspondence with experts was generally slightly higher with Datquant® than with BasGanV2. Indeed, we noted that half of discrepancies with Datquant® derives from false negative cases due to the mis-positioning of the background ROI that sometimes fell partially outside the brain (example in Fig. 3a). On the other hand, we noted that the majority of issues with BasGanV2 comes from a high number of borderline cases in instances read as negative by the experts. This might be due to factors, such as differences in collimators and reconstruction parameters as well as in control composition, that are fixed in the software and cannot be customized, as instead happens for Datquant®. Moreover, in some instances, the fixed ROI of BasGanV2 may overboard the actual boundaries of nuclei, as it is evident in Fig. 2b, leading to falsely reduced average counts and thus to false borderline or even positive results for a normal scan. As a final remark, in rare instances (4 in the present series representing the 2.6%), both software may fail as in the parkinsonian patient of Fig. 3c in whom the right background ROI partly falls outside the brain border with both software.

In conclusion, both Datquant® and BasGanV2 work reasonably well in semi-quantification of DAT SPECT. Both tools have their own strength and pitfalls that must be known in detail by users in order to obtain the best help in visual reading and reporting DAT SPECT.

## Data Availability

The datasets used and/or analyzed during the current study are available from the corresponding author on reasonable request.

## Abbreviations

DAT: Dopamine transporter
MDS-UPDRS-III: Unified Parkinson’s Disease Rating Scale, motor part
PVE: Partial volume effect
P/C: Putamen-to-caudate

## Code availability

Datquant® is a CE registered software with number CE 0459.
